# Structure-based drug design: aiming for a perfect fit

**DOI:** 10.1042/EBC20170052

**Published:** 2017-11-08

**Authors:** Rob L.M. van Montfort, Paul Workman

**Affiliations:** 1 Cancer Research UK Cancer Therapeutics Unit, Division of Cancer Therapeutics, The Institute of Cancer Research, London SM2 5NG, U.K.; 2 Division of Structural Biology, The Institute of Cancer Research, London SW3 6JB, U.K.

**Keywords:** drug discovery and design, pharmacology, structure

## Abstract

Knowledge of the three-dimensional structure of therapeutically relevant targets has informed drug discovery since the first protein structures were determined using X-ray crystallography in the 1950s and 1960s. In this editorial we provide a brief overview of the powerful impact of structure-based drug design (SBDD), which has its roots in computational and structural biology, with major contributions from both academia and industry. We describe advances in the application of SBDD for integral membrane protein targets that have traditionally proved very challenging. We emphasize the major progress made in fragment-based approaches for which success has been exemplified by over 30 clinical drug candidates and importantly three FDA-approved drugs in oncology. We summarize the articles in this issue that provide an excellent snapshot of the current state of the field of SBDD and fragment-based drug design and which offer key insights into exciting new developments, such as the X-ray free-electron laser technology, cryo-electron microscopy, open science approaches and targeted protein degradation. We stress the value of SBDD in the design of high-quality chemical tools that are used to interrogate biology and disease pathology, and to inform target validation. We emphasize the need to maintain the scientific rigour that has been traditionally associated with structural biology and extend this to other methods used in drug discovery. This is particularly important because the quality and robustness of any form of contributory data determines its usefulness in accelerating drug design, and therefore ultimately in providing patient benefit.

The idea to use the information from protein structure to guide the design of new therapeutic agents stems from the 1950s and 1960s when the first protein structures were elucidated by X-ray crystallography [1,2]. Notably, the pioneering work of John Kendrew and Max Perutz in solving the crystal structures of myoglobin and haemoglobin explained the oxygen-carrying/storing properties of these proteins and also shed light on the molecular basis of sickle cell anaemia and at the same time on potential treatments for this disease [3]. Similarly, the determination of the amino acid sequence of insulin by Fred Sanger, and its three-dimensional structure by Dorothy Hodgkin, were used to engineer slowly acting synthetic insulins for the treatment of diabetes [2]. These ground-breaking studies exemplify how therapeutic exploitation has always been closely linked to the fundamental understanding of protein structure and function. Importantly, in addition to valuable insights into protein folds, structure-function relationships and molecular evolution, the availability of the three-dimensional structures of therapeutically relevant proteins also allowed the identification and characterisation of potential inhibitor binding sites and formed the foundation for structure-based drug design (SBDD).

In the 1980s, the field rapidly evolved with biotechnology companies such as Agouron Pharmaceuticals and Molecular Discovery Ltd, respectively, pursuing structure-guided programmes aimed at inhibition of thymidylate synthase for the treatment of cancer, and inhibition of viral neuraminidase to combat influenza [1]. Another early and influential example of this approach was the use of the structure of HIV protease [4,5] in the design of four FDA-approved antiviral protease inhibitors (saquinavir, nelfinavir, indinavir and ritonavir) for the treatment of HIV/AIDS [1]. These discoveries in turn followed earlier work on inhibitors of the aspartic protease renin, which converts angiotensinogen into angiotensin I to regulate blood pressure, using molecular modelling based on structures of fungal pepsins [2].

In addition to enabling drug design, the exponential growth over the years in the number of macromolecular structures deposited in the Protein Data Bank (PDB), which at the time of writing stood at 133,920 (Figure 1), also stimulated the development of sophisticated software packages such as GRID [6], LUDI [7], GOLD [8] and GLIDE [9] to facilitate ‘docking’ of inhibitors into their predicted binding sites as well as to computationally analyse inhibitor binding and inform on further enhancement.

**Figure 1:**
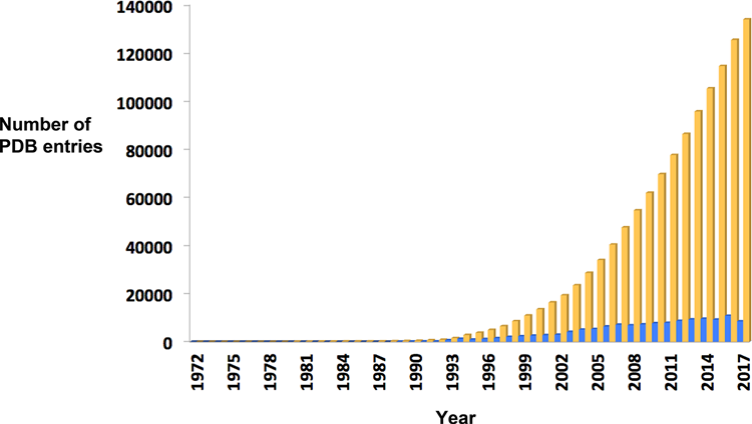
Yearly growth of structures in the Protein Data Bank Beige bars: total number of X-ray, NMR, electron microscopy and modelled structures in the PDB. Blue bars: total number of structures deposited per year. Source: RCSB Protein Data Bank: www.rcsb.org/pdb/home/home.do

In the late 1990s, SBDD continued to evolve and, in particular, was a key enabler of fragment-based drug design (FBDD). This approach has its roots in computational and structural biology research from the 1980s and 1990s with key inputs from basic and applied studies in academia and at companies such Abbott Laboratories, Astex Pharmaceuticals and Vernalis [10–14]. As we write, FBDD has yielded over 30 clinical drug candidates and also three FDA-approved drugs in oncology: the BRAF inhibitor vemurafenib, the BCL-2 inhibitor venetoclax, and the CDK4 inhibitor ribociclib [15]. The main principle underlying FBDD is the efficient sampling of chemical space using small libraries of low-molecular weight (≤250 Da) compounds of low complexity [16], which makes it a complementary approach to the high-throughput screening of large libraries of compounds with lead-like properties including a molecular weight ≤450 Da. Importantly, the small size of fragment libraries and resulting low cost of entry has had the benefit of democratising the use of FBDD in academia and small companies. In addition, FBDD has been incorporated into the tool box of major pharma companies.

Although fragments typically bind with a low potency, they form efficient interactions with the protein target and thus provide attractive starting points for inhibitor design. Nowadays, a plethora of different methods are being used to identify initial fragment hits [17]. They include the traditional biophysical ones such as nuclear magnetic resonance (NMR), surface plasmon resonance (SPR), differential scanning fluorimetry (DSF) — also known as thermal shift assay (TSA), and X-ray crystallography, together with more recent ones like microscale thermophoresis (MST) and mass spectrometry [16,18]. In addition, successful fragment screens have been carried out using *in vitro* biochemical assays [19]. To optimise a fragment hit into a potent lead molecule, a structure-guided approach is critical. Although there are examples in which fragment hits have been optimised using a combination of biophysical and computational methods [20], we are unaware of FBDD campaigns that have not had some contribution from structural data during the hit-to-lead and/or lead optimisation stages of drug discovery.

An area in which structural biology, and hence SBDD, has struggled for many years is that of integral membrane proteins. Generally, these are very difficult to crystallise due to their large hydrophobic areas that are embedded in the membrane and also because their structural and functional integrity is often dependent on the surrounding membrane. However, advances in sample handling, stabilisation, modification and crystallisation, combined with powerful micro-focus synchrotron beamlines, have enabled the structure elucidation of many membrane proteins, including therapeutically relevant ion channels and G-protein coupled receptors (GPCRs), and spawned subsequent SBDD and FBDD approaches for those target classes [21].

The first protein structures have been yielded by exciting new developments such as the X-ray free electron lasers (X-FELs), in which slurries of nanocrystals are irradiated with high-energy femtosecond X-ray pulses allowing the collection of a constructive diffraction image before destruction of the crystal [22,23]. Moreover, simulated experiments suggest that X-ray scattering from single molecules can, in principle, be captured, which could remove the need to crystallise a protein in the future [24].

Parallel developments in electron microscopes, such as the Titan KRIOS, allow the determination of structures of proteins and protein complexes at near-atomic resolution by cryo-electron microscopy (cryoEM). With this technique, the conformation of the protein of interest is again not constrained by a crystal lattice [25]. As this issue of *Essays in Biochemistry* was being finalised, we were delighted at the announcement that three pioneers of cryoEM, Jacques Dubochet, Joachim Frank, and Richard Henderson, were awarded the Nobel Prize in Chemistry. CryoEM structure determination was originally restricted to high-molecular weight complexes (>300 kDa), but the cryoEM structure of isocitrate dehydrogenase 1 shows that structure solution for proteins and complexes smaller than 100 kDa is possible [25]. This achievement brings cryoEM into the realm of SBDD for targets that are not amenable to X-ray crystallography because of their size and/or flexibility.

In addition, cryoEM may provide an easier way to obtain insight into the conformation of a drug target as part of a more physiological protein complex, and thus offer crucial information complementary to the inhibitor design efforts on isolated protein targets or protein domains. Indeed, the first cryoEM structures visualising bound small-molecule ligands have already been reported and include ribosome structures with bound antibiotics [26] and the human 20S proteasome structure with a covalently bound substrate analogue (see Figure 2 and [27]). It will be exciting to see how the cryoEM field can capitalise on these achievements and further develop the technology to make it more routinely applicable to lower molecular weight proteins and with throughputs for iterative use that are more consistent with the demanding timescale of a typical drug discovery project.

**Figure 2:**
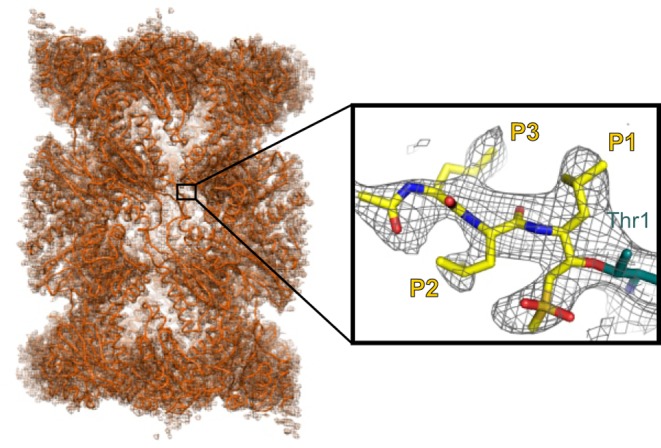
CryoEM structure of the 20S proteasome with bound substrate analogue The left-hand side shows a superposition of the cryoEM density map and 20S proteasome coordinates in orange (PDB accession codes EMD-2981 and 5A0Q). The close-up shows the covalently bound substrate analogue in yellow with the P1, P2 and P3 side chain positions indicated. The density map is displayed in black. Figure kindly provided by Ed Morris.

In this issue of *Essays in Biochemistry*, we have brought together articles that collectively provide a current overview of the field of SBDD and FBDD, and also those that offer an exciting outlook on new developments that advance and complement the current repertoire of methodologies, and are applicable to target classes that are currently not amenable to current approaches.

The issue kicks off with a contribution from Martin Noble and Jane Endicott [28] who describe the use of protein structural data in understanding and interfering with the regulation of cyclin-dependent kinases (CDKs), as examples of a target class that has benefited greatly from structure-based approaches. Other recent examples include highly potent inhibitors of CDKs 8 and 19 which were enabled by SBDD (Figure 3), and that progressed to drug candidates and chemical probes that have seriously questioned the therapeutic window with such agents [29,30].

**Figure 3:**
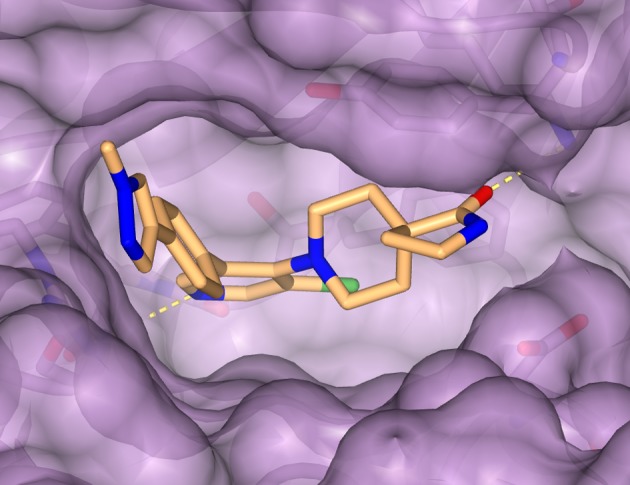
Crystal structure of the chemical tool CCT251545 bound to the CDK8-cyclin C complex The inhibitor CCT251545 is shown in gold, CDK8-cyclin C is displayed in purple and key hydrogen bond interactions are shown in yellow (PDB ID code 5BNJ).

Rod Hubbard and Bas Lamoree [31] then provide a valuable overview of FBDD in which they describe the initial development of methods and ideas in FBDD and give an overview of the current practice and new advances in this field.

Daniel Shiu-Hin Chan et al. [32] describe the use of different mass spectrometry methods in fragment screening and contrast these with the commonly used biophysical methods in FBDD.

Amanda Price, Steven Howard and Benjamin Cons [33] present a detailed example of how a fragment-based approach can be applied to difficult targets such as the KEAP1/NRF2 protein-protein interaction.

Marta Carneiro et al. [34] discuss the valuable contribution that NMR spectroscopy can make to SBDD campaigns and highlight new developments in protein-observed and paramagnetic NMR methods.

Anthony Bradley, Frank von Delft, Chas Bountra and colleagues [35] describe the innovative role that the Structural Genomics Consortium can play in an era where such an abundance of genomics data can present challenges in terms of translation into bespoke drug discovery efforts on novel targets. They also discuss the application of rapid crystallographic fragment screening using the XChem facility at the Diamond Light Source, which has the potential to quickly assess the ‘ligandability’ of a target, and they touch on the importance of structural data in the design of high-quality chemical probes or tool compounds. Furthermore, they describe their concept of the Target Enabling Package (TEP) that provides the research community, via provision of reagents, assays and data, with what they call the often missing link between genetic disease linkage and the development of usefully potent ligands.

In separate contributions, Tom Heightman [36] and Alessio Ciulli [37], together with their respective co-authors, describe the novel PROTAC approach in drug discovery, which is based on VHL (Von Hippel–Lindau)- or cereblon-mediated proteasomal degradation of therapeutics targets, for which structural biology has delivered invaluable insights.

Robert Cheng, Rafael Abela and Michael Hennig [38] provide an exciting insight in how the power of X-ray free electron lasers (X-FELs) can be harnessed to study membrane protein such as GPCRs.

Finally, and very appropriately in view of the recently awarded Nobel Prize mentioned above, Andreas Boland, Leifu Chang and David Barford [39] provide an overview of the current status of cryoEM and offer an insight into the promise and challenges of its application in drug discovery.

Structure-based design is important not only in attaining the goal of discovering drugs that progress to the clinic and regulatory approval for patient benefit, but also in the search for potent, selective and cell-permeant chemical probes that can be used with confidence to explore the mechanistic biology of healthy and diseased cells. Alongside genetic approaches such as RNA interference and CRISPR, such chemical probes can help to validate in a robust way the molecular targets for therapeutic intervention in advance of long and expensive drug-discovery campaigns. Previous reviews have set out both the “promise and perils” of chemical probes [40–43]. Structural biology approaches can help to achieve high levels of potency and selectivity. Moreover, Kevan Shokat has recently stressed the importance, when assessing a new chemical probe (or drug), of the availability of a co-crystal structure demonstrating binding to the target protein and of seeing proof that the activity can be enhanced in a manner that is consistent with the structural model [44]. A recent perspective has emphasized the use and abuse of chemical probes in exploring biology and validating targets for cancer therapy, emphasized the importance of selectivity as part of best practice and stressed the danger to research integrity of using poor quality chemical tools [45].

In addition, on the subject of research integrity, to continue to reliably exploit the rapidly increasing wealth of structural data, as a scientific community, we will have to ensure that the structures that are deposited in public databases such as the PDB are of the highest possible quality. It is worrying that despite the availability of sophisticated crystallographic hardware, data processing and structure refinement software, combined with the current high-quality structure validation tools, we notice an increase in the number of publicly available structures that have been poorly refined, or for which the interpretation borders on wishful thinking. We believe that this originates from a lack of training and/or supervision in the computational and mathematical aspects of structural biology and a limited understanding of protein stereochemistry. A rigorous understanding of crystallographic analyses and crystal structure quality within the scientific community remains critical in appreciating and assessing the value of the structural data deposited in the PDB. The same rigour should be applied to cryoEM structures, many of which are currently determined at resolutions between 5 and 3 Å, a range in which macromolecular structures are relatively poorly defined and ligands often only just about visible. Combined with the fall-off in resolution between the core and the more peripheral parts of cryoEM structures, researchers will have to be extremely careful in the modelling of macromolecular interaction partners and allosteric inhibitors binding to the surface of the studied complex.

We argue that the tradition of scientific rigour within the structural biology community should also extend to biophysical data, especially to complicated but heavily used techniques such as SPR and NMR. For example, a recent investigation by our colleagues into what, based on SPR data, was claimed to be a nanomolar inhibitor of the HSP70 molecular chaperone revealed a non-specific mode of action due to aggregate formation [46]. As scientists we are the guardians of our data — whether this is structural, biophysical, a chemical tool, or any other type of data — and we should remind ourselves of the spirit of the seminal Kleywegt and Jones paper on structure quality [47] and avoid the situation where freedom being given leads to liberties being taken.

The important role that SBDD has taken in modern drug discovery of chemical probes and drugs is exemplified by the increasing trend in some organizations, including our own, to bring together within single unified groups or departments the key related technologies of protein production, biochemical assay development, biophysical assays and X-ray crystallography — in order to maximize the efficiencies in the design and use of protein constructs, and synergies between protein structure and function.

We hope that our selection of articles will serve to educate non-expert readers who are looking to gain a sense of the current status and future prospects of structure-based approaches to discovering new drugs and chemical probes, and at the same time prove to be of interest to the aficionados in the field. It should always be remembered that potency and selectivity are not the only essential properties of drugs. The drive towards these key features must be coupled of course to optimising within the same molecule other critical properties such as selectivity, cell permeability, and pharmacokinetic and pharmacodynamic properties — a challenge that requires some compromises on particular individual features. Nevertheless, we hope that readers will be convinced that by aiming for, and often achieving through the approaches discussed here, a sufficiently close-to-perfect fit for the desired target, the application of structure-based approaches will allow us to further accelerate and enhance the discovery of chemical probes for mechanistic investigations and drugs for patient benefit.

## References

[1] Jaskolski M., Dauter Z. and Wlodawer A. (2014) A brief history of macromolecular crystallography, illustrated by a family tree and its Nobel fruits. FEBS J 281, 3985–4009 24698025 10.1111/febs.12796 PMC6309182

[2] Thomas S.E., Mendes V., Kim S.Y., Malhotra S., Ochoa-Montano B., Blaszczyk M. (2017) Structural biology and the design of new therapeutics: from HIV and cancer to mycobacterial infections: a paper dedicated to John Kendrew. J. Mol. Biol. 429, 2677–2693 28648615 10.1016/j.jmb.2017.06.014

[3] Perutz M.F., Rosa J. and Schechter A. (1978) Therapeutic agents for sickle cell disease. Nature 275, 369–370 692720 10.1038/275369a0

[4] Navia M.A., Fitzgerald P.M., McKeever B.M., Leu C.T., Heimbach J.C., Herber W.K (1989) Three-dimensional structure of aspartyl protease from human immunodeficiency virus HIV-1. Nature 337, 615–620 2645523 10.1038/337615a0

[5] Blundell T. and Pearl L. (1989) Retroviral proteinases. A second front against AIDS. Nature 337, 596–597 2645521 10.1038/337596a0

[6] Goodford P.J. (1985) A computational procedure for determining energetically favorable binding sites on biologically important macromolecules. J. Med. Chem. 28, 849–857 3892003 10.1021/jm00145a002

[7] Bohm H.J. (1992) LUDI: rule-based automatic design of new substituents for enzyme inhibitor leads. J. Comput. Aided Mol. Des. 6, 593–606 1291628 10.1007/BF00126217

[8] Verdonk M.L., Cole J.C., Hartshorn M.J., Murray C.W. and Taylor R.D. (2003) Improved protein-ligand docking using GOLD. Proteins 52, 609–623 12910460 10.1002/prot.10465

[9] Halgren T.A., Murphy R.B., Friesner R.A., Beard H.S., Frye L.L., Pollard W.T. (2004) Glide: a new approach for rapid, accurate docking and scoring. 2. Enrichment factors in database screening. J. Med. Chem. 47, 1750–1759 15027866 10.1021/jm030644s

[10] Jencks W.P. (1981) On the attribution and additivity of binding energies. Proc. Natl. Acad. Sci. U.S.A. 78, 4046–4050 16593049 10.1073/pnas.78.7.4046 PMC319722

[11] Verlinde C.L., Rudenko G. and Hol W.G. (1992) In search of new lead compounds for trypanosomiasis drug design: a protein structure-based linked-fragment approach. J. Comput. Aided Mol. Des. 6, 131–147 1624956 10.1007/BF00129424

[12] Shuker S.B., Hajduk P.J., Meadows R.P. and Fesik S.W. (1996) Discovering high-affinity ligands for proteins: SAR by NMR. Science 274, 1531–1534 8929414 10.1126/science.274.5292.1531

[13] Nienaber V.L., Richardson P.L., Klighofer V., Bouska J.J., Giranda V.L. and Greer J. (2000) Discovering novel ligands for macromolecules using X-ray crystallographic screening. Nat. Biotechnol. 18, 1105–1108 11017052 10.1038/80319

[14] Hartshorn M.J., Murray C.W., Cleasby A., Frederickson M., Tickle I.J. and Jhoti H. (2005) Fragment-based lead discovery using X-ray crystallography. J. Med. Chem. 48, 403–413 15658854 10.1021/jm0495778

[15] Blundell T.L. (2017) Protein crystallography and drug discovery: recollections of knowledge exchange between academia and industry. IUCrJ 4, 308–321 10.1107/S2052252517009241 PMC5571795 28875019

[16] Erlanson D.A., Fesik S.W., Hubbard R.E., Jahnke W. and Jhoti H. (2016) Twenty years on: the impact of fragments on drug discovery. Nat. Rev. Drug. Discov. 15, 605–619 27417849 10.1038/nrd.2016.109

[17] Hubbard R.E. and Murray J.B. (2011) Experiences in fragment-based lead discovery. Methods Enzymol 493, 509–531 21371604 10.1016/B978-0-12-381274-2.00020-0

[18] Qin S., Ren Y., Fu X., Shen J., Chen X., Wang Q. (2015) Multiple ligand detection and affinity measurement by ultrafiltration and mass spectrometry analysis applied to fragment mixture screening. Anal. Chim. Acta 886, 98–106 26320641 10.1016/j.aca.2015.06.017

[19] Silva-Santisteban M.C., Westwood I.M., Boxall K., Brown N., Peacock S., McAndrew C. (2013) Fragment-based screening maps inhibitor interactions in the ATP-binding site of checkpoint kinase 2. PLoS ONE 8, e65689 10.1371/journal.pone.0065689 23776527 PMC3680490

[20] Winter A., Sigurdardottir A.G., DiCara D., Valenti G., Blundell T.L. and Gherardi E. (2016) Developing antagonists for the Met-HGF/SF protein-protein interaction using a fragment-based approach. Mol. Cancer Ther. 15, 3–14 26712116 10.1158/1535-7163.MCT-15-0446

[21] Christopher J.A., Aves S.J., Bennett K.A., Dore A.S., Errey J.C., Jazayeri A. (2015) Fragment and structure-based drug discovery for a class C GPCR: discovery of the mGlu5 negative allosteric modulator HTL14242 (3-chloro-5-[6-(5-fluoropyridin-2-yl)pyrimidin-4-yl]benzonitrile). J. Med. Chem. 58, 6653–6664 26225459 10.1021/acs.jmedchem.5b00892

[22] Boutet S., Lomb L., Williams G.J., Barends T.R., Aquila A., Doak R.B. (2012) High-resolution protein structure determination by serial femtosecond crystallography. Science 337, 362–364 22653729 10.1126/science.1217737 PMC3788707

[23] Barends T.R., Foucar L., Botha S., Doak R.B., Shoeman R.L., Nass K. (2014) De novo protein crystal structure determination from X-ray free-electron laser data. Nature 505, 244–247 24270807 10.1038/nature12773

[24] Neutze R., Wouts R., van der Spoel D., Weckert E. and Hajdu J. (2000) Potential for biomolecular imaging with femtosecond X-ray pulses. Nature 406, 752–757 10963603 10.1038/35021099

[25] Merk A., Bartesaghi A., Banerjee S., Falconieri V., Rao P. and Davis M.I. (2016) Breaking Cryo-EM resolution barriers to facilitate drug discovery. Cell 165, 1698–1707 27238019 10.1016/j.cell.2016.05.040 PMC4931924

[26] Wong W., Bai X.C., Brown A., Fernandez I.S., Hanssen E., Condron M. (2014) Cryo-EM structure of the Plasmodium falciparum 80S ribosome bound to the anti-protozoan drug emetine. eLife 3, e03080 10.7554/eLife.03080 PMC4086275 24913268

[27] da Fonseca P.C. and Morris E.P. (2015) Cryo-EM reveals the conformation of a substrate analogue in the human 20S proteasome core. Nat. Commun. 6, 7573 26133119 10.1038/ncomms8573 PMC4506541

[28] Martin M.P., Endicott J.A. and Noble M.E.M. (2017) Structure-based discovery and development of cyclin-dependent protein kinase inhibitors. Essays Biochem. 61, 000–000, 10.1042/EBC20170040 PMC6248306 29118092

[29] Dale T., Clarke P.A., Esdar C., Waalboer D., Adeniji-Popoola O., Ortiz-Ruiz M.J. (2015) A selective chemical probe for exploring the role of CDK8 and CDK19 in human disease. Nat. Chem. Biol. 11, 973–980 26502155 10.1038/nchembio.1952 PMC4677459

[30] Clarke P.A., Ortiz-Ruiz M.J., TePoele R., Adeniji-Popoola O., Box G., Court W. (2016) Assessing the mechanism and therapeutic potential of modulators of the human Mediator complex-associated protein kinases. eLife 5, e20722 10.7554/eLife.20722 27935476 PMC5224920

[31] Lamoree B. and Hubbard R.E. (2017) Current perspectives in fragment-based lead discovery (FBLD). Essays Biochem. 61, 000–000, 10.1042/EBC20170028 PMC5869234 29118093

[32] Chan D.S.H., Whitehouse A.J., Coyne A.G. and Abell C. (2017) Mass spectrometry for fragment screening. Essays Biochem. 61, 000–000, 10.1042/EBC20170071 28986384

[33] Price A.J., Howard S. and Cons B.D. (2017) Fragment-based drug discovery and its application to challenging drug targets. Essays Biochem. 61, 000–000, 10.1042/EBC20170029 29118094

[34] Carneiro M.G., AB E., Theisgen S. and Siegal G. (2017) NMR in structure-based drug design. Essays Biochem. 61, 000–000, 10.1042/EBC20170037 29118095

[35] Bradley A.R., Echalier A., Fairhead M., Strain-Damerell C., Brennan P., Bullock A.N. (2017) The SGC beyond Structural Genomics: redefining the role of 3D structures by coupling genomic stratification with fragment-based discovery. Essays Biochem. 61, 000–000, 10.1042/EBC20170051 PMC5869235 29118096

[36] Lebraud H. and Heightman T.D. (2017) Protein degradation: a validated therapeutic strategy with exciting prospects. Essays Biochem. 61, 000–000, 10.1042/EBC20170030 28970340

[37] Hughes S.J. and Ciulli A. (2017) Molecular recognition of ternary complexes: a new dimension in the structure-guided design of chemical degraders. Essays Biochem. 61, 000–000, 10.1042/EBC20170041 PMC5869862 29118097

[38] Cheng R.K.Y, Abela R. and Hennig M. (2017) X-ray free electron laser: opportunities for drug discovery. Essays Biochem. 61, 000–000, 10.1042/EBC20170031 29118098

[39] Boland A., Chang L. and Barford D. (2017) The potential of cryo-electron microscopy for structure-based drug design. Essays Biochem. 61, 000–000, 10.1042/EBC20170032 29118099

[40] Workman P. and Collins I. (2010) Probing the probes: fitness factors for small molecule tools. Chem. Biol. 17, 561–577 20609406 10.1016/j.chembiol.2010.05.013 PMC2905514

[41] Frye S.V. (2010) The art of the chemical probe. Nat. Chem. Biol. 6, 159–161 20154659 10.1038/nchembio.296

[42] Bunnage M.E., Chekler E.L. and Jones L.H. (2013) Target validation using chemical probes. Nat. Chem. Biol. 9, 195–199 23508172 10.1038/nchembio.1197

[43] Arrowsmith C.H., Audia J.E., Austin C., Baell J., Bennett J., Blagg J. Nat. Chem. Biol. 11, 536–541 26196764 10.1038/nchembio.1867 PMC4706458

[44] Dang C.V., Reddy E.P., Shokat K.M. and Soucek L. (2017) Drugging the ‘undruggable’ cancer targets. Nat. Rev. Cancer 17, 502–508 28643779 10.1038/nrc.2017.36 PMC5945194

[45] Blagg J. and Workman P. (2017) Choose and use your chemical probe wisely to explore cancer biology. Cancer Cell 32, 268–270 10.1016/j.ccell.2017.07.010 PMC5559281 28810148

[46] Evans L.E., Cheeseman M.D., Yahya N. and Jones K. (2015) Investigating apoptozole as a chemical probe for HSP70 inhibition. PLoS ONE 10, e0140006 26458144 10.1371/journal.pone.0140006 PMC4601772

[47] Kleywegt G.J. and Jones T.A. (1995) Where freedom is given, liberties are taken. Structure 3, 535–540 8590014 10.1016/s0969-2126(01)00187-3

